# Diffusion tensor imaging in unclear intramedullary tumor-suspected lesions allows separating tumors from inflammation

**DOI:** 10.1038/s41393-021-00741-2

**Published:** 2021-12-30

**Authors:** Marc Hohenhaus, Yorn Merz, Jan-Helge Klingler, Christoph Scholz, Ulrich Hubbe, Jürgen Beck, Katharina Wolf, Karl Egger, Marco Reisert, Nico Kremers

**Affiliations:** 1 Department of Neurosurgery, Medical Center - University of Freiburg, Faculty of Medicine, University of Freiburg, Freiburg, Germany; 2 Department of Neurology, Medical Center - University of Freiburg, Faculty of Medicine, University of Freiburg, Freiburg, Germany; 3 Department of Neuroradiology, Medical Center - University of Freiburg, Faculty of Medicine, University of Freiburg, Freiburg, Germany; 4 Department of Radiology, Tauernklinikum Zell am See, Academic Teaching Hospital of the Paracelsus University Salzburg and Medical University of Vienna, Zell am See, Austria; 5 Department of Radiology, Medical Physics, Medical Center - University of Freiburg, Faculty of Medicine, University of Freiburg, Freiburg, Germany

**Keywords:** Spinal cord diseases, Spine structure, Cancer imaging

## Abstract

**Design:**

Prospective diagnostic study.

**Objectives:**

Primary imaging-based diagnosis of spinal cord tumor-suspected lesions is often challenging. The identification of the definite entity is crucial for dedicated treatment and therefore reduction of morbidity. The aim of this trial was to investigate specific quantitative signal patterns to differentiate unclear intramedullary tumor-suspected lesions based on diffusion tensor imaging (DTI).

**Setting:**

Medical Center - University of Freiburg, Germany.

**Methods:**

Forty patients with an unclear tumor-suspected lesion of the spinal cord prospectively underwent DTI. Primary diagnosis was determined by histological or clinical work-up or remained indeterminate with follow-up. DTI metrics (FA/ADC) were evaluated at the central lesion area, lesion margin, edema, and normal spinal cord and compared between different diagnostic groups (ependymomas, other spinal cord tumors, inflammations).

**Results:**

Mean DTI metrics for all spinal cord tumors (*n* = 18) showed significantly reduced FA and increased ADC values compared to inflammatory lesions (*n* = 8) at the lesion margin (*p* < 0.001, *p* = 0.001) and reduced FA at the central lesion area (*p* < 0.001). There were no significant differences comparing the neoplastic subgroups of ependymomas (*n* = 10) and other spinal cord tumors (*n* = 8), but remaining differences for both compared to the inflammation subgroup. We found significant higher ADC (*p* = 0.040) and a trend to decreased FA (*p* = 0.081) for ependymomas compared to inflammations at the edema.

**Conclusion:**

Even if distinct differentiation of ependymomas from other spinal cord neoplasms was not possible based on quantitative DTI metrics, FA and ADC were feasible to separate inflammatory lesions. This may avoid unnecessary surgery in patients with unclear intramedullary tumor-suspected lesions.

## Introduction

Spinal cord tumors (SCT) are rare and surgical intervention is usually required [1, 2]. The neurological status and the histological diagnosis are the most important factors for long-term outcome [2–4]. Complete surgical resection is the primary goal for spinal ependymoma, hemangioblastoma and other non-infiltrating tumors. For infiltrating neoplastic entities, like astrocytic tumors or lymphomas, cautious procedures with biopsy for histological classification and following radiotherapy are primarily recommended. A non-surgical treatment is required for tumor-mimicking inflammatory lesions. An invasive diagnostic procedure or treatment with the risk for neurological deterioration has to be avoided in such cases.

Therefore, reliable presurgical diagnostics are required to achieve optimal functional outcome of affected patients. For imaging-based diagnosis of SCT, magnetic resonance imaging (MRI) is the gold standard, because of its highest lesion perceptibility and anatomic resolution [5, 6]. Conventional acquired T2- and T1-weighted sequences without and with contrast enhancement (CE) are state of the art [2, 7–9]. There are several newer MRI techniques, like MR spectroscopy, Phase-contrast imaging or Diffusion-weighted imaging, but which were mainly applied in studies. They have not entered clinical routine yet, because the precise diagnostic value is often still heterogeneous due to the rather small case series. Some spinal cord lesions present typical signal clusters within the conventional MRI sequences, like hemangioblastomas with usually strong nodular CE, vascularization, associated cysts and surrounding edema, or cavernomas with CE on T1-weighted images and usually blood degradation products within the spinal mass [7]. The characterization of other entities, especially the differentiation of astrocytic tumors and ependymomas, remains still challenging [7, 10, 11]. Diffusion tensor imaging (DTI), a special MRI technique with depiction of tissue diffusion alterations and strengths, was reported to enhance the diagnosis of entity and characteristics of SCT within some case series [12–16], but not yet in larger clinical trials. This effect seems to be based on the different structure of the tumor mass itself as well as the constitution of the surrounding spinal cord tissue [16].

Because of the high relevance for further treatment planning, it is crucial to improve the presurgical imaging diagnostics. We expected to gain an imaging-based differentiation of non-infiltrating ependymomas from other SCT and inflammatory lesions by a specific quantitative DTI parameter configuration.

## Methods

The aim of this prospective, diagnostic study was to investigate specific quantitative signal patterns to classify unclear intramedullary tumor-suspected lesions based on DTI.

### Study population

Patient data were sampled within a prospective, single-center, single-arm cohort study. The study was approved by the local ethics committee (reference 145/14) and reported to the national clinical trial registry (DRKS00008994). We prospectively included patients who presented with an unclear, intramedullary, tumor-suspected lesion through the outpatient clinic or the Emergency Center of the Department of Neurosurgery and Neuroradiology of the University Medical Center Freiburg between 2014 and 2019. All patients gave their written consent. In case of age <18 years, the consent was given by a parent or legal guardian. Patients with radiological clearly definable diagnosis on conventional MRI, like hemangioblastoma, cavernoma or lipoma, were excluded. Additional exclusion criteria were the common MRI contraindications.

### Patient data

Patients’ baseline characteristics and clinical status by modified McCormick Score (mMCS) were documented [17]. There was no defined follow-up procedure for the included patients due to the primary diagnostic imaging setting of the study.

### Evaluation of conventional MRI

According to the usual radiological work-up, conventional T1-weighted sequences in sagittal and transverse plane without and with contrast agent and T2-weighted sequences in sagittal und transverse plane were analyzed in every patient concerning the following common imaging features: none, slight (thickened spinal cord with remaining perimedullary cerebrospinal fluid space) or severe (thickened spinal cord with complete occupation of the spinal canal) space occupation; central or asymmetric intramedullary position; presence of one or multiple associated tumor cysts or intralesional hemorrhages; no, mild (less than two vertebral bodies), moderate (two to five vertebral bodies) or extensive (more than five vertebral bodies) perilesional edema; presence of no, focal nodular, annular or diffuse CE, as well as the occurrence of a syrinx. The evaluation was done independently by one experienced radiologist (NK) and one instructed post-graduate student (YM). Inter-observer variability was calculated and for further evaluation both had to reach consensus.

### Study imaging protocol

All patients received an additional high-resolution, three-dimensional T2-weighted SPACE sequence as anatomical reference and a study-specific DTI sequence using a 3.0T scanner (SIEMENS^®^, Erlangen, Germany). DTI was performed using the RESOLVE sequence (Readout Segmentation Of Long Variable Echo-trains, SIEMENS^®^) with the following imaging parameters: axial acquisition, TR 7000 ms, TE 77 ms, Matrix 110 × 110, slice thickness 2.0 mm, resulting resolution 2 × 2 × 2 mm, *b* values 0 and 1000 s/mm^2^, 20 diffusion directions [18]. From July 2017 to October 2019 an adapted DTI protocol had to be used due to scanner updates with modified parameters: sagittal acquisition, TR 2600 ms, TE 66 ms, Matrix 110 × 200; slice thickness 2.5 mm, resulting resolution 1 × 1 × 2.5 mm; *b* values 0 and 2000 s/mm², 40 diffusion directions. Ten patients were measured with this adapted protocol. Correction for Gibbs artifact, followed by a denoising step was applied for image acquisition [19, 20].

### Evaluation of DTI

**Fig. 1:**
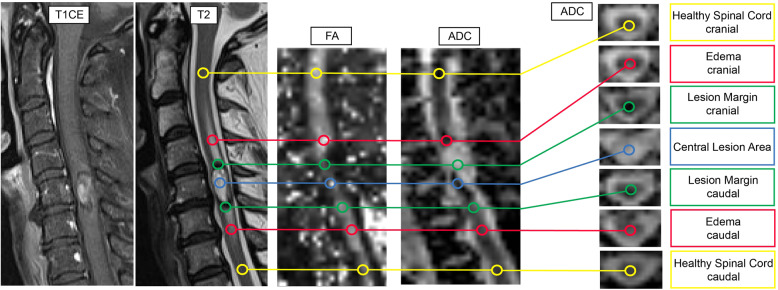
Exemplary determination of the regions of interest (ROI) in a 57-years-old woman with a hemorrhagic ependymoma at level C5 and associated cranial and caudal edema (patient No. 6). ROI setting was done directly on FA and ADC maps with anatomical correlation to 3D T2-weighted images.

The image post-processing was done through the specialized in-house software pipeline NORA (www.nora-imaging.org) and for tensor calculation the following open source toolbox was used: https://www.uniklinik-freiburg.de/mr-en/research-groups/diffperf/fibertools.html. For calculation of the DTI parameters we defined seven regions of interest (ROI) for every tumor-suspected lesion: cranial normal spinal cord, cranial edema, cranial lesion margin, central lesion area, caudal lesion margin, caudal edema, and caudal normal spinal cord. For further analysis we calculated the mean of the cranial and caudal value, if both anatomical locations were available for ROI setting. If there was only a cranial or caudal ROI definable, the single value was used. Principle of ROI setting is depicted in Fig. 1. Spherical ROI were applied with a radius of two voxels placed on the fractional anisotropy (FA) and apparent diffusion coefficient (ADC) maps with corresponding 3D T2-weighted images as anatomical correlation. Within every ROI, the average FA and ADC values were calculated. All ADC values are given in 10^−3^ mm^2^/s.

### Statistics

Data processing and statistical analysis were performed using IBM SPSS Statistics® (Version 25). The inter-observer reliability for the radiographic conventional MRI characteristics was determined by Kappa statistics [21]. Normal distribution was assessed by Shapiro–Wilk-test. *t*-Test for unpaired samples for normally distributed values and Mann–Whitney *U* test for non-normally distributed values were used to compare the different entity groups. Levene’s test was used to assess the equality of variances. *p* value <0.05 was considered statistically significant.

## Results

### Baseline characteristics study population

We prospectively included 40 patients with a mean age of 45.4 (SD 18.3) years. Gender distribution showed 18 male (45.0%) and 22 female (55.0%) patients. Eighteen patients (45.0%) reported pain and/or showed mild neurological deficits like paresthesia or slight gait disturbance (mMCS 1 or 2), whereas 14 patients (35.0%) were severely neurologically deteriorated (mMCS 3–5). Seven patients (17.5%) with a spinal cord lesion showed only non-specific symptoms, like neck pain or dizziness, leading to primary MRI diagnostics. They are stated as mMCS 0. For the 2-year-old child, the mMCS could not be determined.

After inclusion into the study and DTI measurement, 26 of all 40 patients received a definite diagnosis through their clinical diagnostic work-up or following surgical treatment. Eighteen patients (45%) had a histological proven SCT, whereof ten patients (25%) showed an ependymoma and eight patients (20%) another SCT. In eight patients (20%), an inflammatory process could be detected. Therefore, the left 14 patients (35%) remained with an indeterminate diagnosis without a specific treatment, thus receiving imaging and clinical follow-up examinations. The mean follow-up time for patients with an indeterminate lesion was 31.4 (SD 28.0) months, whereas the diagnosis remained still unclear for this whole time period. The clinical course and follow-up time period of every patient with an indeterminate diagnosis was heterogeneous and is attached as Supplementary 1.

According to their definite diagnosis, we grouped our study population in “spinal cord tumors”, including the two subgroups “ependymomas” and “other spinal cord tumors”, as well as “inflammation” and “indeterminate”. The baseline parameters for each group are shown in Table 1.

**Table 1:** Baseline characteristics of all patients and separated for the diagnostic groups.

	All patients	Ependymoma	Other SCT	Inflammation	Indeterminate
Number of patients	40	10	8	8	14
Male: Female	18: 22	6: 4	2: 6	6: 2	4: 10
Age, years, mean (SD)^a^	45.4 (18.3)	52.6 (18.9)	43.5 (25.0)	38.3 (11.6)	45.5 (16.6)
mMCS^b^					
0	7	0	1	0	6
1–2	18	7	3	3	5
3–5	14	3	4	5	2

*SCT* spinal cord tumors, *mMCS* modified McCormick score.

^a^There was no significant difference for age between the four diagnosis groups (*p* = 0.396).

^b^For the 2-year-old child the mMCS could not be determined.

### Conventional MRI

The predominant location of the intramedullary lesions was the cervical spine (*n* = 25, 62.5%), whereof two lesions expanded to the craniocervical junction and four to the thoracic spine. The other lesions were located at the thoracic spine (*n* = 15, 37.5%) with one tumor expanding to the upper lumbar spine. The consensus MRI lesion characteristics of both raters for every patient are shown in Table 2.

**Table 2:** Conventional MRI characteristics of all 40 patients.

No.	Age	Localization	Conventional MRI characteristics							Treatment	Diagnosis
			Space occupation	Intramedullary position	Cysts	Hemorrhage	Edema	Contrast enhancement	Syrinx		
1	62	T6/7	Slightly	Central	Multiple	Yes	No	Diffuse	Yes	Resection	Ependymoma
2	75	T10	Slightly	Central	One	No	Extensive	Nodular	Yes	Resection	Ependymoma
3	80	T2/3	Severely	Central	No	No	Extensive	Diffuse	No	Resection	Ependymoma
4	47	C1/2	Severely	Central	Multiple	Yes	Extensive	Annular	Yes	Resection	Ependymoma
5	67	C3/4	Slightly	Central	Multiple	Yes	Moderate	Nodular	No	Resection	Ependymoma
6	57	C5	Slightly	Central	Multiple	Yes	Moderate	Nodular	No	Resection	Ependymoma
7	43	C5–T2	Severely	Central	Multiple	Yes	Moderate	Diffuse	No	Resection	Ependymoma
8	43	C7/T1	Slightly	Central	No	No	Mild	No	No	Resection	Ependymoma
9	30	C0–3	Severely	Central	No	No	No	Nodular	No	Resection	Ependymoma
10	22	C0–2	Severely	Central	Multiple	No	Moderate	Diffuse	No	Resection	Ependymoma
11	46	T3–7	Severely	Central	Multiple	Yes	Mild	Diffuse	Yes	Biopsy	Anaplastic oligoastrocytoma
12	64	C3/4	Slightly	Central	No	No	Mild	Diffuse	No	Resection^a^	Anaplastic astrocytoma
13	65	T5/6	Severely	Central	No	Yes	Extensive	Diffuse	Yes	Resection	Hemangiopericytoma
14	11	C3–7	Severely	Central	Multiple	No	Mild	Nodular	No	Resection	Pilocytic astrocytoma
15	11	C2/3	Severely	Central	One	No	Mild	Annular	Yes	Resection	Pilocytic astrocytoma
16	38	C1–4	Slightly	Central	Multiple	Yes	Mild	Diffuse	No	Biopsy	Gliotic tumorous process
17	34	T12/L1	Slightly	Asymmetric	No	No	Mild	No	No	Biopsy	Low-grade glioma
18	79	T7/8	Slightly	Central	No	No	Moderate	Diffuse	No	Clinical Diagnostics	B-cell lymphoma
19	33	T3–5	Slightly	Asymmetric	No	No	Moderate	Nodular	No	Biopsy	Acute inflammation
20	47	T4/5	Slightly	Central	No	No	Mild	Annular	No	Clinical Diagnostics	Clinically isolated syndrome
21	23	T5–8	Slightly	Central	No	No	Mild	Annular	No	Clinical Diagnostics	Clinically isolated syndrome
22	35	T7/8	Slightly	Asymmetric	No	No	No	Diffuse	No	Biopsy	Clinically isolated syndrome
23	32	T9	Slightly	Asymmetric	No	No	Mild	Diffuse	No	Clinical Diagnostics	Clinically isolated syndrome
24	58	C3	Slightly	Asymmetric	No	No	No	Diffuse	No	Clinical Diagnostics	Clinically isolated syndrome
25	30	C4	No	Asymmetric	No	No	No	No	No	Clinical Diagnostics	Clinically isolated syndrome
26	48	C1–5	Slightly	Asymmetric	No	No	Extensive	Diffuse	No	Clinical Diagnostics	Postinfectious myelitis
27	32	C1/2	Slightly	Central	No	No	Extensive	No	Yes	Decompression	Indeterminate
28	52	C3/4	Slightly	Central	Multiple	Yes	No	Nodular	No	Conservative	Indeterminate
29	2	C2–7	Severely	Asymmetric	No	No	Moderate	No	No	Conservative	Indeterminate
30	58	C2/3	Slightly	Asymmetric	No	No	No	Diffuse	No	Conservative	Indeterminate
31	35	C3/4	Slightly	Central	Multiple	Yes	No	No	No	Conservative	Indeterminate
32	43	C4	Slightly	Central	No	No	Mild	Nodular	No	Conservative	Indeterminate
33	55	C4	Slightly	Central	No	No	No	Nodular	No	Conservative	Indeterminate
34	41	C4/5	Slightly	Central	One	No	Mild	Diffuse	No	Conservative	Indeterminate
35	56	C5/6	No	Asymmetric	No	No	Moderate	Nodular	No	Conservative	Indeterminate
36	74	C7–T4	Slightly	Asymmetric	No	No	Moderate	No	No	Conservative	Indeterminate
37	48	C7/T1	Severely	Central	No	No	No	Nodular	No	Conservative	Indeterminate
38	56	T3/4	Slightly	Central	No	No	No	No	No	Conservative	Indeterminate
39	45	T2	Slightly	Central	No	No	No	Nodular	No	Conservative	Indeterminate
40	40	T3/4	Severely	Central	No	No	Moderate	Nodular	No	Conservative	Indeterminate

^a^This patient received a resection of the cerebral pathology found in the staging of the complete neuroaxis and additional cerebrospinal fluid sampling, resulting in the diagnosis of an intramedullary metastasis of the cerebral anaplastic astrocytoma.

The absolute agreement of both raters for all seven evaluated lesion characteristics in all 40 patients was 83.2% (233 of in total 280 ratings). By consensus reading, the final decision was adjusted predominantly toward the experienced radiologist in 35 of the 47 disagreed cases (74.5%). The absolute agreement separated for each lesion characteristic and the associated Kappa values were added as Supplementary 2. The inter-observer reliability was “moderate” to “almost perfect” with the following Kappa (*K*) values: Space occupation *Κ* = 0.641 (SD 0.117, *p* < 0.001), intramedullary position *Κ* = 0.827 (SD 0.095, *p* < 0.001), cysts *Κ* = 0.744 (SD 0.091, *p* < 0.001), hemorrhage *Κ* = 0.630 (SD 0.148, *p* < 0.001), edema *Κ* = 0.560 (SD 0.098, *p* < 0.001), CE *Κ* = 0.579 (SD 0.101, *p* < 0.001), syrinx *Κ* = 0.725 (SD 0.150, *p* < 0.001) [21].

### Diffusion tensor imaging

Because of the heterogeneous lesion location and expansion, the ROI setting had to be adapted individually, like mentioned within the methods section. In all patients we could define a ROI at the central lesion area and at the lesion margin. But not every patient showed a perilesional edema or normal spinal cord areas within the field of view. The number of applicable ROI for every patient group is attached as Supplementary 3.

We separated our DTI parameter evaluation for patients with a distinct (*n* = 26) and patients with an indeterminate diagnosis (*n* = 14).

For the central lesion area, SCT showed significantly lower mean FA values compared to the inflammation group (0.249 (SD 0.106) vs. 0.457 (SD 0.081), *p* < 0.001). This significant difference remained at the lesion margin (0.333 (SD 0.128) vs. 0.526 (SD 0.048), *p* < 0.001). The mean ADC values were significantly higher in SCT at the lesion margin (1.487 (SD 0.273) vs. 1.027 (SD 0.241), *p* = 0.001), without reaching statistical significance at the central lesion area (*p* = 0.129). The perilesional edema and the normal spinal cord areas showed no significant differences for both DTI parameters.

**Fig. 2:**
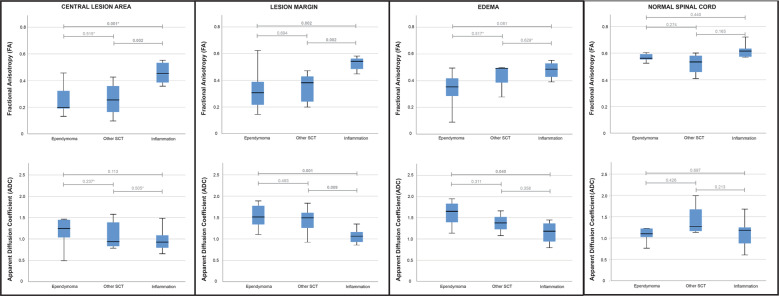
Boxplots for the FA (above) and ADC (below) metrics of all three definite diagnosis groups. SCT = spinal cord tumors. All ADC values are given in 10^−3^ mm^2^/s. *p-values: Group comparison using Mann–Whitney *U* test because of non-normally distributed FA values within the central tumor area for ependymomas and within the edema for other SCTs as well as the ADC values within the central tumor area for other SCTs. For all other group comparisons *t*-test for unpaired samples was applied.

The comparison of the two SCT subgroups revealed no significant differences for all evaluated areas (Fig. 2). Ependymomas showed the lowest mean FA at the central lesion area (0.240 (SD 0.099)) and the lesion margin (0.322 (SD 0.147)) and the highest mean ADC values for both areas (1.300 (SD 0.502) and 1.528 (SD 0.265)) without reaching statistical significance compared to the other SCT (*p* = 0.237–0.694, Table 3 and Fig. 2). For the comparison of both SCT subgroups with the inflammations, the differences remained similar to all SCT together (Fig. 2) for the central and margin lesion areas. For the perilesional edema, a statistically significant difference was only detected for the ADC between ependymoma and the inflammation group (1.600 (SD 0.304) vs. 1.154 (SD 0.282), *p* = 0.040, Fig. 2) with a marginally non-significant trend for the FA (0.355 (SD 0.134) vs. 0.479 (SD 0.069), *p* = 0.081). The normal spinal cord areas showed no significant differences between all groups as expected (Fig. 2).

**Table 3:** DTI measurements at the different evaluated anatomical areas for all four diagnostic groups.

	Fractional anisotropy (FA)				Apparent diffusion coefficient (ADC)			
	Ependymoma	Other SCT	Inflammation	Indeterminate	Ependymoma	Other SCT	Inflammation	Indeterminate
Central lesion area (*n*)	10	8	8	14	10	8	8	14
Mean (SD)	0.240 (0.099)	0.260 (0.120)	0.457 (0.081)	0.295 (0.124)	1.300 (0.502)	1.172 (0.518)	0.970 (0.264)	1.132 (0.520)
Lesion margin (*n*)	10	8	8	14	10	8	8	14
Mean (SD)	0.322 (0.147)	0.347 (0.107)	0.526 (0.048)	0.446 (0.144)	1.528 (0.265)	1.436 (0.293)	1.027 (0.241)	1.062 (0.366)
Edema (*n*)	7	3	4	6	7	3	4	6
Mean (SD)	0.335 (0.134)	0.423 (0.125)	0.479 (0.069)	0.391 (0.161)	1.600 (0.304)	1.375 (0.292)	1.154 (0.282)	1.248 (0.412)
Healthy spinal cord (*n*)	5	4	7	13	5	4	7	13
Mean (SD)	0.567 (0.032)	0.519 (0.083)	0.599 (0.085)	0.515 (0.132)	1.191 (0.403)	1.417 (0.394)	1.101 (0.366)	0.969 (0.295)

All ADC values are given in 10^−3^ mm^2^/s.

*SCT* = spinal cord tumors.

Some data were not normally distributed, therefore the following subgroups were compared by Mann–Whitney *U* test: FA at the central tumor area for ependymomas and at the edema for other SCT, as well as ADC values at the central tumor area for other SCT and patients with indeterminate diagnosis. The average values were given as mean values for the sake of clarity.

**Fig. 3:**
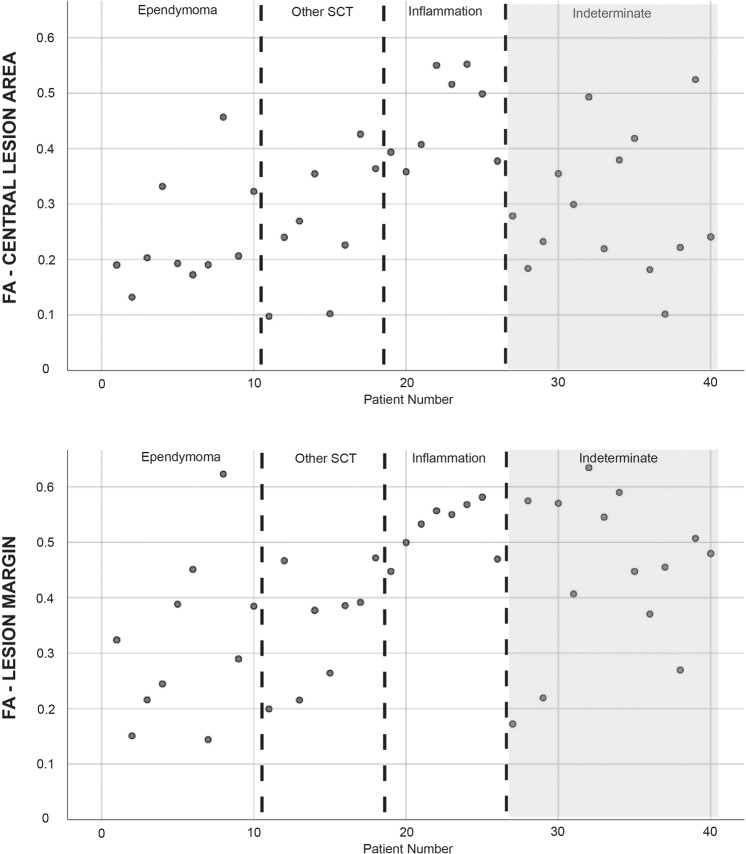
Scatterplots for the individual FA values of all patient groups at the central lesion area and the lesion margin. SCT = spinal cord tumors. The indeterminate diagnosis group is shaded in light gray.

Figure 3 depicts the scatterplots of all 40 evaluated patients for the FA values within the central lesion area and the lesion margin. There was a noticeable clustering from lower FA values in ependymomas to increasing values in other SCT and inflammations (from left to right). The depiction of the indeterminate diagnosis at the right side of the scatterplot (light gray) shows a wide range of FA values from 0.101 to 0.525 for the central lesion area and 0.172 to 0.635 for the lesion margin.

## Discussion

Aim of this study was to detect a specific DTI parameter configuration to separate spinal ependymomas from other SCT and inflammatory diseases. We could demonstrate significantly lower FA and increased ADC values for spinal cord neoplasms compared to inflammatory lesions. Based on our current DTI measurements, a reliable separation of ependymomas from other SCT was not feasible.

This is in line with one of our previous publications showing successful separation of patients with inflammatory lesions due to DTI-based subjective streamline analysis [22]. Inflammatory pathologies presented consistently with normal appearing streamlines in the region of the intramedullary lesion, whereas neoplastic lesions showed displaced or disrupted streamlines. The recent results confirmed the previous data of the subjective streamline analysis.

Pathological diffusion alterations, depicted through abnormal DTI values, are known to reflect different cellular structures within neoplastic as well as the surrounding tissue of the spinal cord. There are some smaller case series showing decreased FA and elevated ADC values in intramedullary astrocytomas compared to healthy spinal cord [13, 23, 24]. This reflects a disturbed structure of the normally high anisotropic white matter of the spinal cord with its parallel orientation of nerve fibers.

The differentiation of the intramedullary tumor entities itself is still challenging. Maj et al. reported a DTI-based differentiation of infiltrating and non-infiltrating SCT in 18 patients [16]. Significant higher FA values were detected for non-infiltrating ependymomas (*n* = 12) compared to infiltrating astrocytic tumors (*n* = 6) at the peritumoral margins (0.399 (SD 0.08) vs. 0.304 (SD 0.1), *p* = 0.007) and peritumoral edema (0.439 (SD 0.11) vs. 0.350 (SD 0.1), *p* = 0.029). There was no significant FA difference within the tumor mass itself (0.205 (SD 0.1) vs. 0.228 (SD 0.1), *p* = 0.470). The ADC values were significantly higher within the tumor mass for non-infiltrative tumors (1.59 (SD 0.55) vs. 1.12 (SD 0.2), *p* = 0.016) but without a significant difference for the peritumoral margin or edema. They postulated higher tumor cellularity in the peritumoral zone of infiltrating tumors to be responsible for the FA decrease. Setzer et al. differentiated 14 intramedullary lesions preoperatively based on Diffusion Tensor Tractography as resectable or non-resectable depending on the appearance of the fiber structure at the lesion [14]. Quantitative DTI metrics were not reported within this work.

In our study, FA values within the tumor mass as well as at the tumor margin seem to be lowest among ependymomas and to increase in other tumor types and to be the highest in inflammatory lesions. The scatterplot of the individual FA values at the central lesion area and lesion margin of all patients (Fig. 3) shows an increase from left (ependymomas) to right (inflammations). There was one outlier (patient No. 8) among the ependymoma group with a FA > 0.4 (central lesion area) and FA > 0.5 (lesion margin). Unfortunately, this patient showed an unclear artifact cranial of the tumor mass (see Supplement 4), possibly affecting also the distant measured DTI parameters. Seven of all ten ependymomas showed a FA ≈ 0.2, indicating a strong disorganized diffusion at the central lesion area. The other SCT showed FA values from 0.1 (pilocytic astrocytoma and anaplastic oligoastrocytoma) to 0.43 (low-grade glioma of the conus medullaris). The patients of the inflammation group showed FA values >0.35 (mean 0.457 (SD 0.081)) within the lesion, nearest to the values of the normal spinal cord (mean 0.545 (SD 0.106)). This corresponds to a minor diffusion alteration and seems to be due to the predominantly acute inflammatory response but basically intact fiber structure in those patients [25]. Even at the lesion margin the mentioned effects continue, whereas the spreading of the FA values for the ependymomas seems to increase (Fig. 3). Artifacts or intralesional hemorrhages might disturb this signal presentation. In summary, there is a trend to lower FA signals in ependymomas, possibly with a specific value around 0.2 at the tumor mass itself.

Liu et al. evaluated the difference between several SCT (*n* = 12, a combination of astrocytomas, ependymomas and glioblastomas) and tumor-like lesions (*n* = 13, inflammatory lesions with multiple sclerosis, transverse myelitis or sarcoidosis) [26]. The tumor-like lesions showed higher FA (0.39 (SD 0.111) vs. 0.232 (SD 0.076), *p* = 0.002) and lower ADC (0.862 (SD 0.166) vs. 1.285 (SD 0.505), *p* = 0.016) values than the SCT within the lesion, which is in line with our findings. Additionally, there was also a clear deterioration of the diffusion strength compared to normal spinal cord segments. The baseline values at the normal appearing spinal cord within our cohort were comparable with values reported in literature and therefore reliable (FA: mean 0.545 (SD 0.106) and ADC: mean 1.101 (SD 0.360)) [16, 27].

Overall, there are some former reports of a reproducible differentiation of neoplastic lesions from healthy spinal cord and inflammatory lesions, which could be confirmed throughout our results. We might postulate, that a primarily unclear tumor-suspected lesion with a FA > 0.4 or ADC ≈ 1.0 is suggestive for an inflammatory disease without indication for surgery or biopsy. Grounding on the pathophysiology, the inflammatory lesions might have a less strong disarranged diffusion compared to the normal spinal cord because of the potential integrity of the tissue structure. This has a relevant clinical impact, because of the non-invasive differentiation of neoplastic and non-neoplastic lesions with their primary conservative treatment. There are still several reports of spinal cord biopsies in inflammatory lesions, like even in our cohort (No. 19 and 22) [28, 29]. This is a risk for neurological deterioration and has to be avoided.

There are different areas of interest for the evaluation of DTI metrics in a tumor-suspected lesion. We detected alterations at all anatomical sites: the central lesion area as well as at the lesion margin and the associated edema. Maj et al. showed significant differences at the peritumoral margin and edema for the FA and at the tumor mass for the ADC [16]. For the differentiation of neoplastic and inflammatory lesions, the lesion margin showed consistently significant differences for both parameters, FA and ADC, whereas at the central lesion area only revealed significances for the FA. Therefore, the margin of the suspected lesion seems to be the most relevant area for evaluation.

Fourteen of all included patients remained without definite diagnosis (“indeterminate”). Due to no (six patients) or minor neurological deterioration (three patients with mMCS 1 and two patients with mMCS 2) or declining surgical treatment, these patients underwent follow-up examinations. The DTI metrics for this patient group showed values ranging throughout the whole spectrum as depicted in Fig. 3 in light gray (FA values 0.101–0.525 for the central lesion area and 0.172–0.635 for the lesion margin, mean (SD) shown in Table 3). We suspect, that correspondingly all possible entities are reflected within this group. Unfortunately, we received no definite diagnosis for precise evaluation.

A major limitation, also within our work, is the low incidence of SCT resulting in a small sample size for mono-centric studies. This restriction can only be solved by larger multi-center trials or (inter-) national register studies. Another limitation seems to be the heterogeneous dimension of the tumor-suspected lesions. Tumors with a large space occupation or enormous edema might show more heterogeneous signal clusters than smaller lesions. Side effects like intratumoral hemorrhages, a large syrinx or even signal artifacts (like patient No. 8) may affect the lesion characterization. Additionally, the lesion location could be a relevant attribute. Lesions in the upper cervical spine might show slightly different DTI values than in the lower thoracic spine, because of the different anatomical constitution of the spinal cord. There are reports concerning diverging normal values between the cervical and thoracic spine [27]. Besides localization, the widely ranging age of all included patients (range 2–80 years, mean 45.4 (SD 18.3) years) can be a confounder too, whereas an age-dependency of DTI parameters is reported in the literature for measurements of the healthy spinal cord [30–33]. There are no reports of age-dependent alterations within tumorous areas so far, but an impact of patients’ age could be not be addressed by our data. In principle, larger age- and gender-matched studies would be preferable, but due to the rare pathology of SCT this would reduce the evaluable sample size considerably.

Another limitation of our study is due to the DTI application in clinical routine. For the conventional evaluation of all spinal cord lesions, the high-resolution 3D T2 SPACE was used. The anatomical resolution of Diffusion-weighted MR images is notoriously not as good as for T1- or T2-weighted sequences, whereas a precise discrimination of tissue alterations on Diffusion-weighted images is limited. This complicates the evaluation of the DTI parameters when setting the ROI, because of the small anatomical structures dealing with the spinal cord. In principle, the highest image resolution would be desirable for anatomical discrimination as well as for the reduction of partial volume effects. But in clinical routine, there has to be always the compromise between the acquisition time of the study imaging, that must be tolerable for the patient, and an adequate resolution to address the aimed question. The applied resolution was chosen similarly to the reports within the literature [14–16, 26]. Another complicating factor is, that there is no conformity concerning the most reproducible and reliable ROI size and setting procedure. We chose a ROI size of two voxels, which was smaller than the previously described in the literature in order to avoid measuring surrounding structures to increase precision. The ROI were set directly at the DTI maps to avoid anatomically distortions. Another imaging limitation of this evaluation is the change of the MRI protocol, which was due to scanner updates after the measurement of 30 patients.

At the moment, there is still no radiological technique that reliably separates the different intramedullary tumor entities. For the future, we probably need an integration of different imaging resources with conventional MRI, DTI, MR spectroscopy or new innovative sequences in combination with international studies or registries, to gain a better non-invasive differentiation of intramedullary tumors.

## Conclusions

We conclude, that even though a distinct differentiation of ependymomas and other SCT was not possible due to objective DTI metrics, a separation of inflammatory lesions was feasible in our study cohort based on FA and ADC values. This may avoid unnecessary surgery in patients with unclear intramedullary tumor-suspected lesions. To achieve better tumor differentiation, higher case numbers are needed, requiring larger (inter-) national registry studies.

## Supplementary information

Supplementary information

## Data Availability

The datasets analyzed during the current study are available from the corresponding author on reasonable request.
