# Supplementary material for: Diffusion tensor imaging in unclear intramedullary tumor-suspected lesions allows separating tumors from inflammation
Source: Spinal Cord. 2021 Dec 30;60(7):655–63. doi: 10.1038/s41393-021-00741-2 (PMC9287173; doi:10.1038/s41393-021-00741-2)
Supplement: Supplementary file 1 — Supplementary information [file 41393_2021_741_MOESM1_ESM.docx]

| **No.** | **Age**  (years) | **Localisation** | **mMCS** | **Follow-up time** (month) | **Treatment procedure** |
| --- | --- | --- | --- | --- | --- |
| 27 | 32 | C 1/2 | 3 | 57 | Surgical decompression and dura extension plastic without spinal cord biopsy with regressive syrinx within the MRI follow-ups for two years and clinical stable disease |
| 28 | 52 | C 3/4 | 2 | 12 | Clinical progressive and MRI stable disease till the end of the study,  recommended surgery denied by the patient up to now |
| 29 | 2 | C 2-7 | n.a. | 72 | Clinical and MRI stable disease |
| 30 | 58 | C 2/3 | 0 | 62 | Clinical and MRI stable disease |
| 31 | 35 | C 3/4 | 0 | 32 | Clinical and MRI stable disease |
| 32 | 43 | C 4 | 0 | 0 | No further clinical or MRI follow-up examination at our department |
| 33 | 55 | C 4 | 0 | 13 | Clinical and MRI stable disease |
| 34 | 41 | C 4/5 | 0 | 20 | Clinical and MRI stable disease |
| 35 | 56 | C 5/6 | 2 | 29 | Clinical symptom regression to mMCS 1 with only slightly sensory deficits without pain and MRI stable disease |
| 36 | 74 | C 7-T 4 | 3 | 51 | Clinical symptom regression (sensory deficits/pain) during a parallel immunoglobulin therapy because of a chronic-inflammatory demyelinating polyneuropathy (CIDP) and small-fiber neuropathy with MRI stable disease |
| 37 | 48 | C 7/T 1 | 0 | 0 | No further clinical or MRI follow-up examination at our department |
| 38 | 56 | T 3/4 | 1 | 0 | No further clinical or MRI follow-up examination at our department |
| 39 | 45 | T 2 | 0 | 0 | No further clinical or MRI follow-up examination at our department |
| 40 | 40 | T 3/4 | 1 | 12 | Clinical stable but MRI progressive disease,  recommended surgery denied by the patient up to now |

**Supplement 1 - Clinical course and follow-up time of every patient with an indeterminate diagnosis.** n.a. = not available because of the low patients age (reduced motoric strength of the left arm in side-by-side comparison from birth).

|  | Number (%) of patients without agreement | Kappa (SD) |
| --- | --- | --- |
| **Space occupation** | 7/40 (17.5) | 0.641 (0.117) |
| **Intramedullary position** | 3/40 (7.5) | 0.827 (0.095) |
| **Cysts** | 5/40 (12.5) | 0.744 (0.091) |
| **Hemorrhage** | 5/40 (12.5) | 0.630 (0.148) |
| **Edema** | 13/40 (32.5) | 0.560 (0.098) |
| **Contrast enhancement** | 11/40 (27.5) | 0.579 (0.101) |
| **Syrinx** | 3/40 (7.5) | 0.725 (0.150) |

**Supplement 2 - Overview of observer disagreement separated for all evaluated tumor characteristics and the associated Kappa (SD) values.**

|  |  | Ependymoma | Other SCT | Inflammation | Indeterminate |
| --- | --- | --- | --- | --- | --- |
| Central lesion area (n) |  | 10 | 8 | 8 | 14 |
| Lesion margin (n) | Cranial | 7 | 7 | 8 | 14 |
|  | Caudal | 10 | 7 | 8 | 13 |
|  | Total | 10 | 8 | 8 | 14 |
| Edema (n) | Cranial | 4 | 3 | 4 | 6 |
|  | Caudal | 7 | 3 | 4 | 4 |
|  | Total | 7 | 3 | 4 | 6 |
| Healthy spinal cord (n) | Cranial | 2 | 3 | 6 | 13 |
|  | Caudal | 5 | 3 | 7 | 12 |
|  | Total | 5 | 4 | 7 | 13 |

**Supplement 3 - Number of applicable ROI for all anatomical locations and all evaluated patient groups.** We calculated the mean value if a ROI at the cranial and caudal lesion margin, edema or normal spinal cord was available. If there was only one cranial or one caudal ROI definable, the single value was used. SCT = Spinal Cord Tumors.


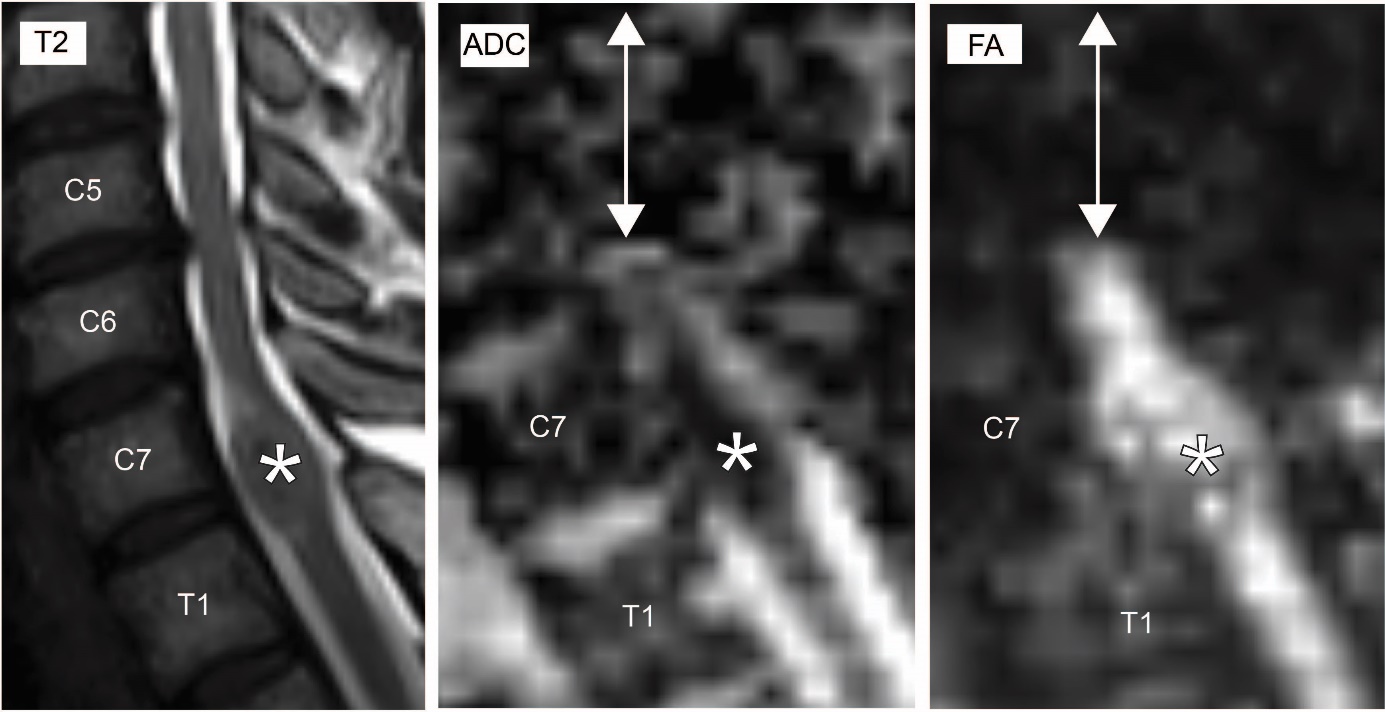


**Supplement 4** - Images of a 43 years old patient (No. 8) showing a spinal cord tumor (*) with an associated slightly edema at C7/T1. The patient received complete tumor resection with a histologically proven ependymoma. There was an unclear artifact on the evaluated B0 images as well as on the associated ADC and FA maps (white arrow). The artifact begins at the level of vertebral body six and continues cranially, so that normal spinal cord, edema and cranial tumor margin cranial of the spinal cord lesion could not be sufficiently analyzed. ROI were applicable at the central lesion area and at the caudal lesion margin, edema and normal spinal cord. The artifact should not affect these ROI, which is why the patient was kept in the evaluation.
